# Electrostatic Gelatin Nanoparticles for Biotherapeutic Delivery

**DOI:** 10.3390/gels10120757

**Published:** 2024-11-23

**Authors:** Connor Tobo, Avantika Jain, Madhushika Elabada Gamage, Paul Jelliss, Koyal Garg

**Affiliations:** 1 Biomedical Engineering Department, Saint Louis University, Saint Louis, MO 63103, USA; connor.tobo@slu.edu; 2 Pharmacology and Physiology Department, Saint Louis University, Saint Louis, MO 63104, USA; avantika.jain@slu.edu; 3 Chemistry Department, Saint Louis University, Saint Louis, MO 63103, USA; madhushika.elabadagamage@slu.edu (M.E.G.); paul.jelliss@slu.edu (P.J.)

**Keywords:** gelatin, nanoparticles, electrostatic, extracellular vesicles

## Abstract

Biological agents such as extracellular vesicles (EVs) and growth factors, when administered in vivo, often face rapid clearance, limiting their therapeutic potential. To address this challenge and enhance their efficacy, we propose the electrostatic conjugation and sequestration of these agents into gelatin-based biomaterials. In this study, gelatin nanoparticles (GNPs) were synthesized via the nanoprecipitation method, with adjustments to the pH of the gelatin solution (4.0 or 10.0) to introduce either a positive or negative charge to the nanoparticles. The GNPs were characterized using dynamic light scattering (DLS), X-ray diffraction (XRD), Fourier-transform infrared spectroscopy (FTIR), and Transmission electron microscopy (TEM) imaging. Both positively and negatively charged GNPs were confirmed to be endotoxin-free and non-cytotoxic. Mesenchymal stem cell (MSC)-derived EVs exhibited characteristic surface markers and a notable negative charge. Zeta potential measurements validated the electrostatic conjugation of MSC-EVs with positively charged GNPs. Utilizing a transwell culture system, we evaluated the impact of EV-GNP conjugates encapsulated within a gelatin hydrogel on macrophage secretory activity. The results demonstrated the bioactivity of EV-GNP conjugates and their synergistic effect on macrophage secretome over five days of culture. In summary, these findings demonstrate the efficacy of electrostatically coupled biotherapeutics with biomaterials for tissue regeneration applications.

## 1. Introduction

Mesenchymal stem cells (MSCs) support tissue formation, blood vessel growth, suppress inflammation, and prevent fibrosis [1,2]. MSCs exert these actions by releasing a diverse array of biological products like extracellular vesicles (EVs), cytokines, and growth factors [3]. Because of these benefits and their involvement in the wound healing process, MSCs are widely used as a regenerative therapy [4]. However, they are also prone to a multitude of challenges such as their difficulty to acquire, tumorigenesis, and heterotopic tissue formation [5,6]. EVs are secreted by mammalian cells, including MSCs, for intercellular communication [7]. They are formed by a process in which endocytic vesicles form multivesicular bodies that are subsequently fused with the plasma membrane [8]. Like the plasma membrane, it is known that EVs carry a negative surface charge [9,10]. Following their release, EVs can be transported throughout the body in various fluids, including plasma, saliva, and cerebrospinal fluid [11]. EV communication with target cells includes cellular uptake mechanisms such as phagocytosis, micropinocytosis, cell membrane fusion, and via ligand-receptor interactions depending on the target cell type [12,13]. After entering the target cell, EVs deliver their contents that include bioactive molecules such as lipids, proteins, mRNAs, and microRNAs unique to the cell of origin [13,14,15]. Thus, MSC EVs recapitulate the broad therapeutic effects attributed to MSCs [16,17]. As an acellular MSC byproduct, EVs can readily circulate through organs, elicit a minimal immune response, and stimulate cellular responses [18]. Despite their potential for stimulating regeneration, the administration of EVs in vivo results in rapid clearance and off-target accumulation [19,20]. Therefore, the use of EVs as a treatment for various pathologies, including cancer or tissue injury, is promising, but their effectiveness is hindered by a poor retention time [21]. The delivery method for any treatment, including biological cargo, is paramount for ensuring the effectiveness of the therapeutic. Nanoscale delivery methods offer many advantages due to their size, customizable surface, and multi-functionality, which make them ideal candidates for the delivery of biotherapeutics like EVs [22]. This work investigates nanoparticles capable of electrostatic conjugation with EVs to achieve their retention at the delivery site [23,24,25].

To develop a delivery system for EVs, we focus on gelatin, a commonly used component of food and pharmaceutical products alike [26]. It is a widely studied delivery vehicle for small biomolecules because of its many biological advantages [27]; it contains many binding sites that can be modified or cross-linked, it can be easily processed into a variety of implantable biomaterials, and it is biocompatible, biodegradable, and non-immunogenic [28]. It can be prepared by two different methods from two main animal sources (i.e., mainly porcine and bovine) [29]. Collagen hydrolysis and thermal denaturing steps are preceded by treatment in either acidic conditions or alkaline conditions to yield either type A gelatin or type B gelatin, respectively [26,28]. Alkaline conditions cause deamidation of asparagine and glutamine to yield more aspartic acid and glutamic acid residues on type B gelatin and, thereby an increased amount of carboxylic acid as compared to type A gelatin [26,28]. Thus, functional groups on the surface of gelatin include amino (-NH_2_) and carboxyl (-COOH) groups [30,31]. Chemical conditions can alter the equilibrium between amino/carboxyl groups and gelatin-NH_3_^+^/gelatin-COO^−^ groups [31]. These groups are covalently bound on the gelatin network and can be used to alter the charge of gelatin by either lowering or increasing pH, which would increase the number charged -NH_3_^+^ or -COO^−^ groups, respectively [31]. Hence, type A gelatin treated in acidic conditions would feature an increased number of positively charged -NH_3_^+^ groups while type B gelatin treated in alkaline conditions would feature more negatively charged -COO^−^ groups. The presence of these functional groups also allows for gelatin to be cross-linked [32]. Nanoscale protein carriers share many of the advantageous qualities of gelatin including biocompatibility, biodegradability, and non-immunogenicity [33]. They are also more stable in biological fluids, and this is advantageous for a sustained release of deliverable treatments [34]. The variety of amino acids that make up gelatin, like positively charged lysine and arginine and negatively charged glutamic and aspartic acids, make it a viable option as a nanocarrier that can deliver a variety of drugs or other molecules [33]. Gelatin also features a multitude of amino acids on its chain, like methionine and valine, that are lipophilic and make parts of the chain hydrophobic [35]. These many advantages lead to gelatin nanoparticles being a viable option as a nanocarrier for delivering a variety of drugs and biological molecules.

This work seeks to enhance the therapeutic efficacy of EVs by extending their retention within the delivery matrix, such as a hydrogel/bioscaffold. This is achieved by electrostatically binding EVs to oppositely charged gelatin nanoparticles (GNPs). Given the variability in EV size, we anticipate two primary forms of nanocomplexes: either GNPs will create a protective nanoshell around EVs, or they will cluster in 1:1 or 1:2 configurations with EVs. These electrostatic interactions are expected to increase particle size, slow down diffusion-driven release, and provide physical shielding from a potentially harsh immune environment.

We hypothesized that bioscaffolds encapsulating EV conjugated GNPs would offer a stable and targeted delivery system for tissue engineering applications. Specifically, the electrostatic interaction of EVs with GNPs and their subsequent encapsulation within the gelatin hydrogels will: (1) improve the bioavailability of the EVs by enhancing the number of EVs that can be carried and released at the target site and (2) ensure stability and protection of the electrostatically bound EVs from enzymes, antibodies, and immune cells.

## 2. Results and Discussion

### 2.1. GNP Size, Morphology, and Zeta Potential Characterization

**Figure 1:**
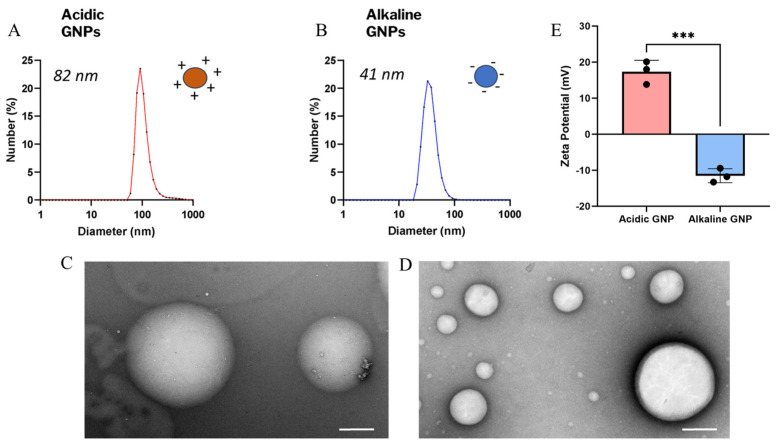
GNP size, morphology, and zeta potential characterization. The size distribution of acidic (**A**) and alkaline (**B**) GNPs is shown. TEM images show particle morphology and integrity of acidic (**C**) and alkaline (**D**) GNPs (scale bar = 200 nm). (**E**) Comparison of zeta potential of acidic GNPs vs. alkaline GNPs is presented. Data reported as mean ± standard deviation. A *t*-test demonstrates statistical significance (*** denotes *p* < 0.001, n = 3).

Dynamic light scattering revealed a mean size of ~82 nm for acidic GNPs (Figure 1A) and ~41 nm for alkaline GNPs (Figure 1B). The variation in particle size with pH has been previously reported for gelatin nanoparticles [36]. Figure 1C,D demonstrate intact and spherical morphology of GNPs post-sonication via TEM imaging. Zeta potential of acidic GNPs was recorded to average 12.76 mV for acidic GNPs and −13.8 mV for alkaline GNPs (Figure 1E).

Table 1 shows DLS and zeta potential measurements taken for particles suspended in nuclease-free water (NF H_2_O), sodium chloride (NaCl), and 1X PBSfollowing the same process. The magnitude of particle zeta potential is decreased following suspension in buffers containing ions. However, the zeta potential of GNPs synthesized in acidic conditions remains positive, while the zeta potential of GNPs synthesized in alkaline conditions remains negative.

### 2.2. X-Ray Diffraction & Fourier-Transform Infrared Spectroscopy Characterization

**Figure 2:**
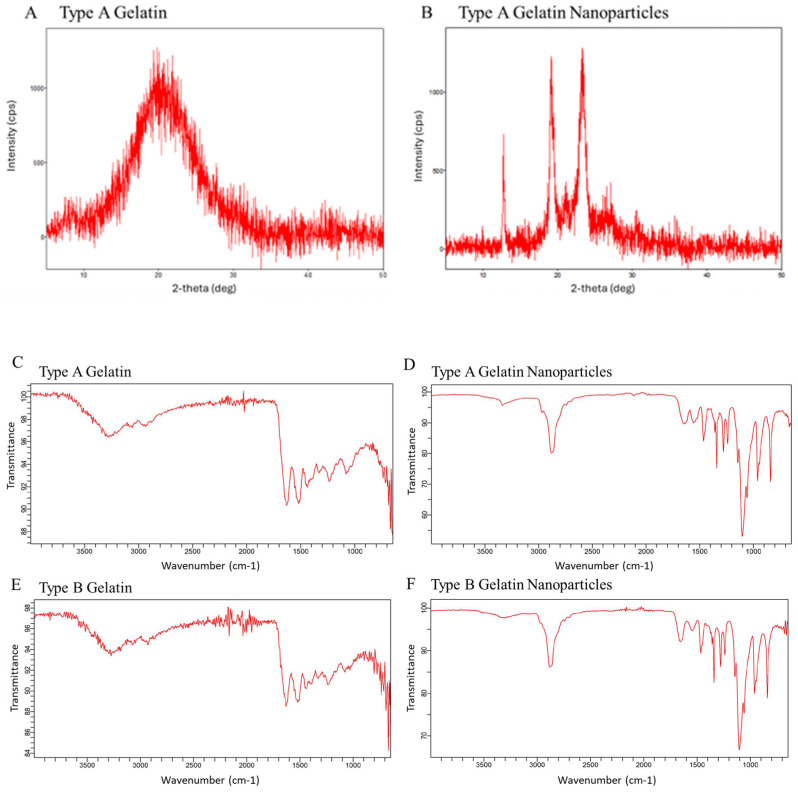
Powder XRD spectra comparing stock type-A gelatin (**A**) and acidic GNPs (**B**). FTIR spectra of both (**C**,**E**) type A and B gelatin and (**D**,**F**) type A and type B GNPs.

XRD curves reveal differences in crystallinity between stock type A gelatin and acidic GNPs (Figure 2A,B). Multiple sharply defined peaks represent the presence of crystalline structure in GNPs, while no defined peaks in gelatin indicate a lack of crystallinity. This increase in crystallinity can be attributed to the rearrangement of the gelatin chains in an ordered fashion following nanoprecipitation and crosslinking, resulting in the development of crystallites in the nanoparticles [37]. This difference demonstrates that the GNP synthesis protocol yields more well-organized particles with a different chemical structure than that of stock gelatin. The increased presence of charged groups affiliated with GNPs, like NH_3_^+^, are critical in mediating crystalline morphology [31]. Bands characteristic of gelatin are shown on FTIR spectra (Figure 2C,E) at 3260 cm^−1^ (amide A, N–H stretching vibrations of –NH_2_ and O–H stretching), 2920 cm^−1^ (amide B, C–H stretching), 1640 cm^−1^ (amide I, C=O stretching), 1540 cm^−1^ (amide II, N–H bending), 1440 cm^−1^ (–CH_2_ bending), and 1180 cm^−1^ (amide III, C–N and N–H Stretching) [38]. Anti symmetric –CH stretching and symmetric stretching of –CH_3_, –CH_2_ and =C–H, correlate to absorption bands at 2939 and 3082 cm^−1^, respectively. The region close to 3381 cm^−1^ features bands that relate to an antisymmetric NH_2_ stretch of the primary amide of gelatin. This region also indicates physically adsorbed water denoted by O–H stretching [39]. The gelatin nanoparticles FTIR spectra (Figure 2D,F) showed a sharper and higher-intensity peak for the Amide A band around 3260 cm^−1,^ indicating dehydration during nanoparticle formation. Amide I and II regions showed lower intensity in the nanoparticles suggesting changes in structure due to crosslinking of gelatin chains following nanoparticle formation.

### 2.3. Endotoxin Content Assessment

An enzymatic assay based on a lysate from washed amebocytes was used to assess endotoxin content. GNP endotoxin content levels were measured below the U.S. Pharmacopeia maximum endotoxin unit (EU) clinical standard of 0.25 EU/mL (Table 2) [40]. Furthermore, disinfecting GNPs with UV light resulted in ~2-fold lower endotoxin content than untreated GNPs. Based on this data, UV-treated GNPs will be used for all in vitro experiments.

### 2.4. Cytotoxicity Assessment

**Figure 3:**
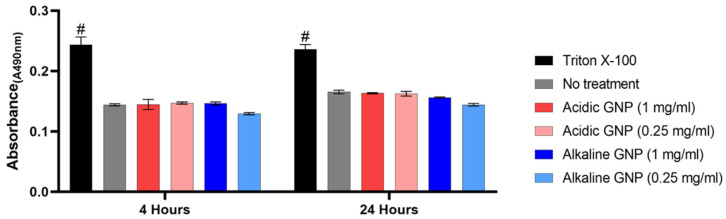
LDH Assay assessing cytotoxicity. No statistically significant differences were detected between GNP treatment groups and untreated media control (# denotes significance between all other groups, *p* < 0.001 (n = 3)).

An LDH cytotoxicity assay measured the degree of cytotoxicity of GNPs. The assay is based on LDH release from cellular cytoplasm into the culture medium from dead or damaged cells. Absorbance measurements (λ = 490 nm) following the assay reaction revealed that GNPs are not cytotoxic (Figure 3).

LDH release by MSCs cultured with both acidic and alkaline GNPs in various concentrations was statistically equivalent to MSCs cultured in complete growth media with no treatment. Treating cells with Triton X-100 for total cell lysis resulted in statistical significance compared to all other groups. These comparisons imply that GNP treatment is not cytotoxic. Cells also appeared visually healthy with no changes in morphology.

### 2.5. EV Size and Surface Marker Characterization

**Figure 4:**
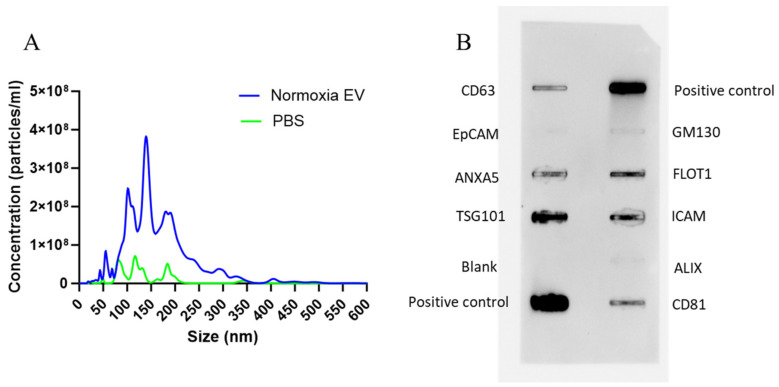
MSC-EV size and surface marker characterization. (**A**) NTA data shows an average EV particle size range of 50–300 nm (n = 5). NTA for 1X PBS is also shown as negative control (n = 3). (**B**) An exo-check array reveals the presence of EV-specific markers, confirming the successful isolation of EVs. A marker for cellular contamination (GM130) was negative.

**Figure 5:**
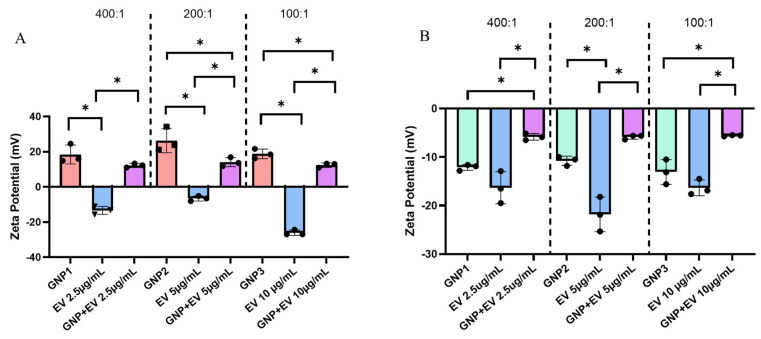
(**A**) Zeta potential measurements comparing acidic GNPs and EVs versus GNP^+^:EV conjugates. From left to right, GNP^+^:EV ratios tested include 400:1, 200:1, and 100:1. Each GNP and conjugate measurement maintained a consistent GNP concentration of 1.0 mg/mL while EV concentration was changed between groups. Different EV concentrations are specified (x-axis). (**B**) The zeta potential of alkaline GNPs, EVs, and their respectively combined solutions in the same ratios of 400:1, 200:1, and 100:1. Statistical comparisons between measurements of different GNP^+^ to EV ratios are not shown for ease of viewing. Data is represented as mean ± standard deviation (n = 3, statistical significance is denoted by * for *p* < 0.05 between groups for One-way ANOVA).

EVs isolated from murine bone marrow MSCs were characterized by NTA and an Exo-Check Exosome Antibody Array (System Biosciences) to determine the size and presence of EV-specific markers, respectively. The size of isolated EVs varied in a range of 50–300 nm (Figure 4A). This size variation is not alarming as EVs are a biological product that may see size variation under different conditions such as cellular confluency, isolation procedure, etc., and EVs are known to exist in a wide size range [41]. An exo-check array also revealed the presence of specific surface and internal markers such as CD81, TSG101, and CD63 (Figure 5B). It also established a lack of cellular contamination by confirming no detection of GM130.

### 2.6. GNP-EV Conjugation and Zeta Potential

Zeta potential measurements confirm the negative zeta potential of EVs as well as reiterate the positive charge of acidic GNPs. It is previously known that EVs carry a negative surface charge [9]. After combining negatively charged EVs with a GNP^+^ suspension, the combined suspension demonstrates a reversal of charge from negative to positive but still less than that of GNPs alone (Figure 5A). This result is consistent at three different ratios of GNP^+^:EV combination. We selected concentration ratios of GNP: EV at 400:1, 200:1, and 100:1 to evaluate the extent of surface coating of EVs by GNPs. We aimed to identify the concentration threshold at which EVs are fully covered with GNPs and the point at which no or partial coverage is observed. This approach was guided by the heterogenous size distribution of EVs ranging from 50–300 nm. Therefore, we speculated that larger EVs would require a higher number/concentration of GNPs for complete coverage, while smaller EVs may reach complete coating at lower ratios. The reversal to a positive charge lends to the conclusion that GNPs are electrostatically conjugated to EVs forming a nanoshell of positively charged particles at all concentrations tested. Figure 5B shows the same experimental setup, but the GNPs used were synthesized in alkaline conditions and possess a negative zeta potential.

The solution combining both alkaline GNP^−^s and EVs demonstrates a drop in zeta potential, indicating a change in surface interactions. Furthermore, these results show that the zeta potential of the combined suspension is not a mere average of the two individual suspensions’ zeta potential. The results in Figure 5 also highlight the variability in the measured zeta potential values of EV samples at different concentrations. Several factors may cause fluctuations in zeta potential measurements of EV samples at varying concentrations. These include inherent heterogeneity in EV size, which can lead to sampling errors due to uneven representation of the population. Additionally, particle–particle interactions change at different concentrations, impacting the measured potential. Variations in the density and distribution of surface charges on the lipid bilayer membrane of EVs may also contribute to the measured zeta potential values.

### 2.7. Release of GNP^+^:EV Conjugates and Their Temporal Effects on Bioactivity

**Figure 6:**
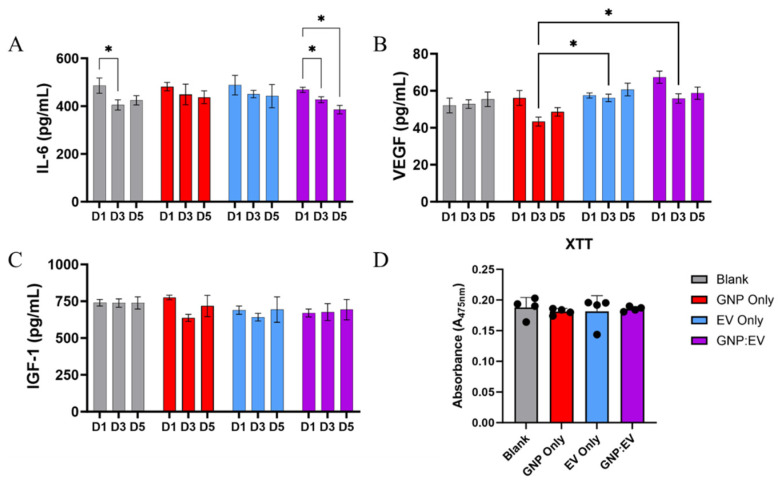
Release of GNP^+^:EV Conjugates and Their Temporal Effects on Bioactivity. (**A**) IL-6 secretion is significantly reduced in groups treated with GNP^+^:EV conjugates over the course of both three and five days, and it is also reduced on day three for cells cultured with control gels (**B**) Macrophages treated with EVs and GNP^+^:EV conjugates secreted significantly more VEGF than cells treated with gels containing only GNPs. (**C**) IGF-1 concentration is not significantly different amongst any groups. (**D**) No differences in cellular proliferation were noted between groups (n = 4, * denotes *p* < 0.05, a repeated measures two-way ANOVA was used for analysis).

To assess the biologic effects of GNP^+^s, EVs, and electrostatically conjugated GNP^+^:EV, were encapsulated inside gelatin gels and placed in the upper chamber of a transwell plate. LPS-stimulated macrophages were cultured in the lower chamber of the transwell. Cell-culture supernatants collected on days 1, 3, and 5 were used to quantify the release of trophic factors (Figure 6). In groups treated with GNP^+^:EV conjugates, IL-6 secretion showed a progressive decline over 5 days of culture. However, the levels of IL-6 were maintained in the GNP and EV groups over time, suggesting that their combination uniquely drives this outcome. Unexpectedly, IL-6 levels also decreased on day three as compared to day one in the control group. However, there is no significant difference in IL-6 secretion when comparing blank gels to other groups (Figure 6A).

VEGF secretion from macrophages was decreased in the GNP^+^ loaded gelatin gel group on day three compared to EV-loaded and GNP^+^:EV-loaded gel groups (Figure 6B). These results suggest that GNPs, but not EVs, reduce VEGF secretion from macrophages on day three, and that the interaction between GNP and EV modulates the release of VEGF differently. Finally, the release of IGF-1, a pro-regenerative growth factor [42], is not affected by any treatment type (Figure 6C).

### 2.8. Discussion

Interest in developing biodegradable nanoparticles, particularly as efficient drug delivery systems, has increased significantly in recent years. Gelatin-based nanoparticles have gained attention as a promising biodegradable carrier for delivering therapeutics due to their biocompatibility [34,43,44,45]. The benefits of using nanoparticles in drug delivery stem from two key properties. First, their small size enables them to pass through narrow capillaries and be absorbed by cells, facilitating effective drug concentration at targeted sites [43,46]. Research suggests that particles with diameters of 100 nm or smaller achieve longer circulation times and may be cleared by macrophages [47]. In this work, we have developed and characterized both cationic and anionic GNPs. Our results show that the GNPs are below 100 nm in size and are stable and non-cytotoxic. To showcase that GNPs can be conjugated with oppositely charged biotherapeutic agents, we demonstrate that cationic GNPs can be electrostatically coupled with MSC-EVs and loaded in gelatin hydrogels for potential immunomodulatory effects.

A multitude of techniques exist for synthesizing GNPs. These include desolation, coacervation-phase separation, emulsification, self-assembly, and nanoprecipitation [48,49,50,51]. Nanoprecipitation is advantageous due to its simple and concise nature compared to other synthesis techniques [52]. Acidic GNPs showed an average hydrodynamic size of 82 nm and an average zeta potential of 12.76 mV, while alkaline GNPs were 41 nm with a zeta potential of −13.8 mV. Both pH treatment types yielded nanoscale particles with opposite surface charges due to the presence of amino (-NH_2_) and carboxyl (-COOH) groups that were able to either accept or donate protons for customizable surface charge [30,31]. XRD and FTIR data revealed gelatin and GNP spectra that showed differences in both crystallinity and emission. GNP endotoxin content was below the US Pharmacopoeia standard, and GNPs in various concentrations were not cytotoxic.

EVs were isolated and characterized to exist on a hydrodynamic size range of 50–300 nm. They were also measured to feature a negative zeta potential regardless of concentration, consistent with pre-existing literature, which has shown that EV zeta potential can reach −20 mV or less [9,10]. Cellular uptake of GNPs and EVs was experimentally confirmed using confocal microscopy. Following characterization of both acidic GNPs and EVs, a novel delivery technique was explored. Currently, EVs have proven difficult to deliver in vivo due to rapid circulatory clearance [19,20]. Despite this limitation, intravenous injection of EVs is currently the most commonly applied delivery method [53]. Visualization and in vivo tracking have demonstrated the availability of intravenously injected EVs in a murine model lasted only 4 h with a half-life of around 2 min [19]. Recently, hydrogels have shown potential as a method for delivery of EVs to target tissue sites. Both Shi et al. and Zhang et al. were able to successfully improve tissue recovery by loading chitosan hydrogels with isolated EVs [54,55]. Both showed improved angiogenesis resulting from the coupling of EV and hydrogel therapies. However, no current literature demonstrates EV delivery either paired with nanoparticles or from a hydrogel aided by nanoparticles. EVs are commonly used themselves as a nanoparticle delivery device [56], but this does not address the established difficulties of effective delivery and prolonged availability.

In this work, we proposed an electrostatic conjugation approach that would leverage the negative surface charge of EVs by conjugating them with GNPs that had been customized with a positive surface charge. We were able to show a reversal from negatively charged EV zeta potential to a positive zeta potential in a suspension of both positively charged GNPs with negatively charged EVs that had been allowed to incubate and electrostatically attach. Subsequently, we tested a combination therapy in vitro featuring EVs conjugated with GNPs encapsulated in a gelatin hydrogel composed of gelatin at a concentration of 6% wt./vol with a mesh size (i.e., average distance between two polymer chain junctions) of 11 nm [57]. This sequestration technique was applied by synthesizing gelatin hydrogels containing GNP^+^:EVs within them. This mesh size of the hydrogel is smaller than the average size of GNPs, EV, and, thus, GNP^+^:EV conjugates. It was hypothesized that treatments would be released as the gelatin gels degraded over time, and the GNP^+^:EV conjugates were released at the slowest rate due to their increased net particle size. Macrophages exposed to GNP^+^:EV-loaded hydrogels in transwell plates showed potential for the combined approach to regulate cytokine secretion over time. Specifically, inflammatory signaling was decreased by GNP^+^:EV-loaded hydrogels by continuously downregulating IL-6 secretion over the course of five days. While the literature offers mixed results on the effects of various types of EVs on IL-6 secretion, MSC-derived EVs have been previously shown to inhibit IL-6 cytokine production, benefitting acute inflammation as a result of liver tissue injury [58].

While promising, this study has several limitations. Firstly, a limitation of this study is that the interaction of GNP and EVs could not be confirmed through other techniques, such as TEM. Therefore, this study only includes findings from zeta potential measurements and bioactivity assays, which provide indirect but valuable insight into the interactions between GNP and EVs. Future studies should explore other alternative imaging methods or techniques to visualize these interactions. Secondly, this study only focuses on one EV dosage for in vitro experiments [53]. For example, Zhang et al. [55] loaded their chitosan hydrogel with 100 µg of EVs for a concentration of 500 µg/mL for in vivo studies. In contrast, our gelatin hydrogel included a much lower dose. Future studies will analyze multiple dosages, including higher dosages suitable for animal experiments. The goal of this work was to establish a proof-of-concept, providing preliminary evidence for the treatment’s viability and setting the foundation for subsequent dose-response analysis. While further experimentation needs to be performed to test alternative EV dosages, this novel technique offers a safe and biocompatible option for biological cargo that is susceptible to biodegradation [23].

## 3. Conclusions

**Figure 7:**
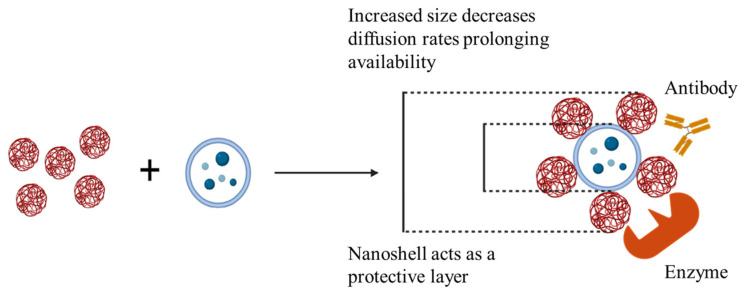
GNP^+^:EV conjugation increases particle size and offers protection for improved retention of biological cargo.

We have synthesized positively and negatively charged GNPs and showed that cationic or positively charged GNPs can be electrostatically conjugated to negatively charged EVs. These GNPs are non-cytotoxic and are endotoxin-free. They are also capable of being phagocytosed by cells. Based on zeta potential changes following combination with biological cargo in suspension, GNPs have the capacity to conjugate with charged therapeutic particles for improved availability by increasing net particle size as well as offering protection from a harsh immunological environment (Figure 7). These conjugates showed potential for both short-term upregulation of pro-regenerative growth factor secretion and downregulation of inflammatory cytokine secretion over time.

Ultimately, our GNP^+^:EV conjugation method offers a multitude of advantages that can render strategies like covalent attachment or chemical conjugation inadequate. These advantages include the preservation of cargo due to simple synthesis, a lack of potentially harmful modification, and maintained biocompatibility after loading [23,59]. Future studies will determine the efficacy of GNP-conjugated EVs in modulating inflammation in a tissue injury rodent model.

## 4. Materials and Methods

### 4.1. Gelatin Nanoparticle (GNP) Synthesis

**Figure 8:**
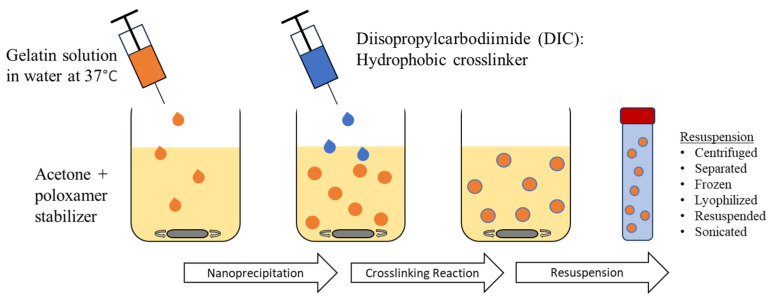
GNP synthesis using the nanoprecipitation method and carbodiimide crosslinking prior to resuspension.

GNPs were prepared using the nanoprecipitation method and chemically crosslinked. Type A or type B gelatin in either acidic or alkaline conditions was subjected to nanoprecipitation to obtain GNPs of different surface charges. To yield positively charged GNPs, 80 mg of type A gelatin powder (G2500, Sigma-Aldrich, St. Louis, MO, USA) was dissolved in autoclaved DI water at 37 °C. The pH of the solution was adjusted to 4 with HCl (1 M). To synthesize negatively charged GNPs, 80 mg of type B bovine skin gelatin (G9382, Sigma-Aldrich, St. Louis, MO, USA) was treated with sodium hydroxide (NaOH) until a pH of 10 was reached. The treated GNP solution was added to a 60% acetone solution containing 6.3 mL acetone, 4.3 mL autoclaved DI water, and 4.5 mL 10% poloxamer-188 solution (P556, Sigma-Aldrich, St. Louis, MO, USA or 6230A, Mirus Bio, Madison, WI, USA), a protein stabilizer. The gelatin solution was added, dropwise, using a 21-gauge syringe needle while the acetone solution was stirring using a stir bar at 600 rpm. Diisopropylcarbodimiide (DIC) (A19292.30, ThermoFisher Scientific, Waltham, MA, USA), a hydrophobic zero-length crosslinker, was added at a final concentration of 9.2 mg/mL. This solution was vortexed and then allowed to stir for 24 h at room temperature to undergo complete crosslinking. The GNPs were then isolated by centrifugation at 5000 RPM for 10 min. This caused a phase separation in which GNPs could be collected from the top layer. The collected GNPs were then frozen, lyophilized, resuspended in sterile nuclease-free water, and sonicated prior to characterization. The nanoprecipitation and crosslinking procedure are shown in Figure 8.

### 4.2. Dynamic Light Scattering (DLS), Transmission Electron Microscopy (TEM) and Zeta Potential Characterization

GNP powder was weighed and added to sterile nuclease-free water to create a 1.0 mg/mL solution. This solution was sonicated on ice to prevent particle aggregation and potential heat-induced degradation. This process is repeated to obtain suspensions of both acidic GNP (GNP^+^) and alkaline GNP (GNP^−^) suspensions. The GNP suspensions were analyzed by DLS using a Zetasizer Nano ZS (Malvern Instruments, Westborough, MA, USA) to determine their hydrodynamic size and zeta potential. At least 3 replicate measurements were taken per sample. DLS and zeta potential are measured by acquiring variables based on scattering of light by particles in a liquid suspension [60,61]. The same zetasizing process was utilized to characterize GNPs’ hydrodynamic size and surface charge in a multitude of buffers. Samples also underwent (TEM) imaging following a negative stain to observe particle morphology. Stock gelatin and GNP powder each underwent XRD (MiniFlex600, Rigaku, Woodlands, TX, USA).

### 4.3. Assessment of GNP Endotoxin Content

A Limulus Amebocyte Lysate (LAL) Assay (Lonza) was used to assess GNP endotoxin content. GNPs underwent UV light sterilization using a 59S UVC LED Sterilizing Box. Samples were exposed to UVC light with an average irradiance of 2145 µW/cm^2^ to 105 µW/cm^2^ at a vertical distance from the light source of 0 cm to 3.7 cm, respectively, within the chamber. Exposure continued for three chamber cycles each consisting of three minutes. Non-UV sterilized GNPs were exposed to open air for the same amount of time. Each group was then resuspended in sterile nuclease free water within a sterile microcentrifuge tube creating 2.5 mg/mL suspensions. Tubes incubated for one hour at 37 °C. UV sterilized and non-UV sterilized GNPs each underwent the LAL assay measuring both kinetic and endpoint data.

### 4.4. Assessment of GNP Cytotoxicity

GNP Cytotoxicity was assessed using a Lactate Dehydrogenase (LDH) assay (Caymen Chemical). Bone marrow-derived murine MSCs (ATCC, passage 5) were cultured in a 48-well plate at densities of 20 k cells/well and 10 k cells/well. As per kit instructions, Triton X-100 (10%) was added to positive control wells to induce total cell lysis. Negative control wells received no additives to their media. GNP treatment groups included both acidic and alkaline GNPs in concentrations of 1.0 and 0.25 mg/mL. 20 µL of GNP suspensions were added to each treatment well. This resulted in an estimated GNP:cell ratio of approximately 10^6^:1 for the 1.0 mg/mL groups. Cell culture supernatants were collected for the LDH assay at 4 h and 24 h after GNP treatment administration. As per kit instructions, cell culture supernatants were incubated with LDH reaction solution for 30 min at 37 °C and the absorbance of the solution was measured at 490 nm.

### 4.5. EV Isolation and Characterization

Murine bone-marrow-derived MSCs (passage ~5) were cultured to confluency in a 75cm^2^ tissue culture flask, rinsed with 1Xphosphate buffered saline (1XPBS), and allowed to incubate overnight in media supplemented with exosome-free FBS (Gibco). The following morning, the media was collected and centrifuged at 3000× *g* for 15 min. EVs were then isolated using an ExoQuick-TC ULTRA EV Isolation Kit (System Biosciences, Palo Alto, CA, USA) according to the manufacturer’s instructions. Following isolation, EV sample protein concentration was measured using a nano-drop spectrophotometer (Thermo Scientific 2000c Spectrophotometer, Wilmington, DE, USA) and analyzed for size and particle concentration using nanoparticle tracking analysis (NTA).

### 4.6. Assessment of GNP and EV Interactions

The zeta potential of three 1.0 mg/mL acidic GNP suspensions and three EV suspensions at 2.5, 5, and 10 µg/mL was measured individually. Then, each EV sample was combined with a 1.0 mg/mL GNP sample and allowed to incubate for 10 min at room temperature. This resulted in three combined suspensions with GNP^+^:EV ratios of 400:1, 200:1, and 100:1 (e.g., 1 mg/mL GNP: 5 µg/mL EV is a ratio of 200:1). The zeta potential of each combined suspension was then measured. This protocol was repeated to perform the same measurements on suspensions combining EVs with alkaline-treated GNPs. EV and GNP concentrations and combination ratios remained consistent in both experiments.

### 4.7. Structural Analysis of Gelatin and Gelatin Nanoparticles (GNPs)

Stock gelatin and GNP powder each underwent X-ray diffraction analysis (XRD) analysis (MiniFlex600, Rigaku, Woodlands, TX, USA). Briefly, samples were placed in a flat sample holder and analyzed at diffraction angles between 0° and 50°. Fourier-transform infrared spectroscopy (FTIR) analysis was performed on a Cary 630 FTIR machine (Agilent Technologies, Santa Clara, CA, USA). To prepare samples for FTIR spectral analysis, stock gelatin and GNP powder were each combined with potassium bromide in a mass ratio of 1:4, respectively. This combined powder was compressed into a thin pellet for analysis. 

### 4.8. Assessing Bioactivity of GNP^+^:EV Conjugates 

Macrophages (RAW264, passage 4, ATCC) were cultured in a tissue culture plastic flask (75 cm^2^) and again stimulated with LPS (100 ng/mL) overnight to induce activation. Activated macrophages were plated in a 24-well plate at a density of 600,000 cells per well in RPMI media (Thermo-Fisher Scientific, Life Technologies Corporation, New York, NY, USA) supplemented with 10% exosome-free fetal bovine serum (Gibco) and 1% penicillin-streptomycin. The cells were allowed three hours to attach and confirm survival. During this three-hour period, gelatin hydrogels were synthesized directly into transwells with treatments integrated into the synthesis process. Test groups include gels without treatment, acidic GNPs only, EVs only, and GNP^+^:EV conjugates. To synthesize gels, 4 mL of sterile water was brought to 50 °C. Within a biosafety cabinet, 420 mg of type A gelatin was sterilized for three cycles in the 59S UVC LED sterilization chamber. In the biosafety cabinet, the gelatin was added to the sterile water and maintained at a temperature of 50 °C while preparing to add the treatments to the gel. Gels were created by combining 200 µL of heated gelatin solution, 50 µL of a crosslinker solution containing EDC and NHS in sterile nuclease-free water, and 100 µL of ‘treatment solution’. This yielded a 6% wt./vol type A gelatin gel crosslinked with EDC at a final concentration of 45.7 mg/mL and NHS at a final concentration of 10.5 mg/mL. It is important to note that a gelatin hydrogel with a gel concentration of 6% wt./vol is known to feature a mesh size (i.e., average distance between two polymer chain junctions) of 11.0 nm [57]. The treatment solution used for control gels was nuclease-free water. The treatment solution for gels synthesized with acidic GNPs was only 10 mg/mL acidic GNP suspension. The EV-only gel treatment solution was a 25 µg/mL EV suspension. Finally, the treatment solution for gels containing GNP^+^:EV conjugates was made by combining 50 µL of 50 µg/mL EV suspension and 50 µL of 20 mg/mL acidic GNP suspension. This yielded a 100 µL solution of both 25 µg/mL EVs and 10 mg/mL GNPs that was allowed to incubate at room temperature for 10 min to allow for conjugation before being combined with the gelatin and crosslinker solutions to create the GNP^+^:EV gels. Gel-loaded transwells were added to wells on day 0. Media was collected and replaced every 24 h between days 1, 3, and 5. The collected media was aliquoted and frozen for bioactivity ELISAs (Peprotech), including Insulin-like growth factor 1 (IGF-1), VEGF, and IL-6. Following media collection on day 5, an XTT cellular proliferation assay (R&D systems) was performed on all wellsas per manufacturer’s instructions. This assay is based on the reduction of the tetrazolium salt, XTT or 2,3-Bis(2-methoxy-4-nitro-5-sulfophenyl)-2H-tetrazolium-5-carboxanilide.

### 4.9. Statistical Analysis

Data are presented as a mean  ±  standard error of the mean. *t*-tests and one-way or two-way analysis of variance were used to determine if there was a significant interaction or main effect between variables. A Tukey’s or Fisher’s LSD post-hoc comparison was utilized to identify the source of significance with *p* < 0.05 unless otherwise specified in figure captions. GraphPad Prism 10.10 for Windows was used to perform statistical analysis and graphing of data.

## Figures and Tables

**Table 1:** Size and zeta potential measurements of acidic and alkaline GNPs suspended in various buffers. Hydrodynamic size and zeta potential are reported as average ± standard deviation.

Suspension	pH	Concentration	Avgerage Size (nm)	Average PDI	Average Zeta Potential (mV)
NF H_2_O	Acidic	1.0 mg/mL	81.84 ± 14.29	0.50	12.76 ± 1.17
	Alkaline	1.0 mg/mL	41.42 ± 26.18	0.51	−13.80 ± 0.29
NaCl	Acidic	1.0 mg/mL	71.96 ± 29.61	0.50	11.70 ± 1.04
	Alkaline	1.0 mg/mL	15.93 ± 8.48	0.59	−3.54 ± 0.27
PBS	Acidic	1.0 mg/mL	13.82 ± 6.18	0.49	2.52 ± 0.68
	Alkaline	1.0 mg/mL	150.21 ± 45.40	0.49	−3.17 ± 1.07

**Table 2:** The endotoxin content for GNPs with and without UV treatment was compared with sterile nuclease-free water (vehicle), a LAL assay blank solution, and a LAL assay positive control solution provided in the kit.

Sample	EU/mL (Mean ± STD)
Nuclease Free Water	0.0145 ± 0.001
LAL Assay Blank	0.012 ± 0.001
UV Treated GNPs	0.0515 ± 0.006
No UV Treatment GNPs	0.1125 ± 0.008
LAL Assay Positive Control	0.6445 ± 0.002

## Data Availability

Data is available upon reasonable request.
